# Plasmonic Ag-Decorated Few-Layer MoS_2_ Nanosheets Vertically Grown on Graphene for Efficient Photoelectrochemical Water Splitting

**DOI:** 10.1007/s40820-020-00512-3

**Published:** 2020-08-25

**Authors:** Dong-Bum Seo, Tran Nam Trung, Dong-Ok Kim, Duong Viet Duc, Sungmin Hong, Youngku Sohn, Jong-Ryul Jeong, Eui-Tae Kim

**Affiliations:** 1 Department of Materials Science and Engineering, Chungnam National University, Daejeon, 34134 Republic of Korea; 2 Department of Chemistry, Chungnam National University, Daejeon, 34134 Republic of Korea

**Keywords:** Photoelectrocatalysis, Molybdenum disulfide, Graphene, Surface plasmon resonance

## Abstract

A controllable approach that combines surface plasmon resonance and two-dimensional (2D) graphene/MoS_2_ heterojunction has not been implemented despite its potential for efficient photoelectrochemical (PEC) water splitting. In this study, plasmonic Ag-decorated 2D MoS_2_ nanosheets were vertically grown on graphene substrates in a practical large-scale manner through metalorganic chemical vapor deposition of MoS_2_ and thermal evaporation of Ag. The plasmonic Ag-decorated MoS_2_ nanosheets on graphene yielded up to 10 times higher photo-to-dark current ratio than MoS_2_ nanosheets on indium tin oxide. The significantly enhanced PEC activity could be attributed to the synergetic effects of SPR and favorable graphene/2D MoS_2_ heterojunction. Plasmonic Ag nanoparticles not only increased visible-light and near-infrared absorption of 2D MoS_2_, but also induced highly amplified local electric field intensity in 2D MoS_2_. In addition, the vertically aligned 2D MoS_2_ on graphene acted as a desirable heterostructure for efficient separation and transportation of photo-generated carriers. This study provides a promising path for exploiting the full potential of 2D MoS_2_ for practical large-scale and efficient PEC water-splitting applications.

**Electronic supplementary material:**

The online version of this article (10.1007/s40820-020-00512-3) contains supplementary material, which is available to authorized users.

## Highlights

- Controllable and large-scale practical growth of plasmonic Ag-decorated vertically aligned 2D MoS_2_ nanosheets on graphene.
- Realization of the synergistic effects of surface plasmon resonance and favorable graphene/MoS_2_ heterojunction to enhance the photoelectrochemical reactivity of 2D MoS_2_.

**Electronic supplementary material:**

The online version of this article (10.1007/s40820-020-00512-3) contains supplementary material, which is available to authorized users.

## Graphical abstract

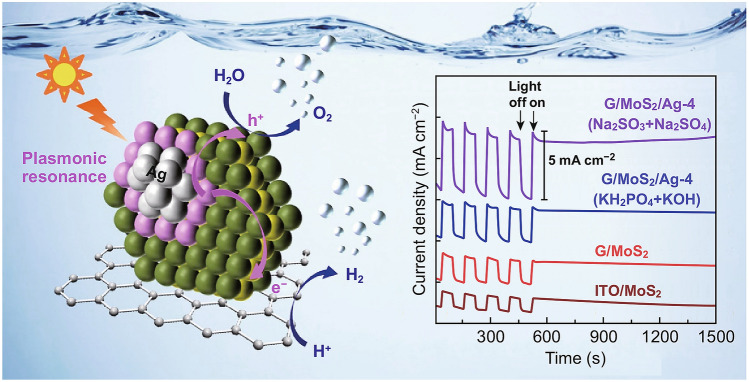

## Introduction

Photoelectrochemistry (PEC) and photocatalysis of semiconductors have been extensively studied as effective approaches for energy conversion, such as hydrogen gas production by water splitting, and for environmental applications, such as air/water purification, water disinfection, and hazardous waste remediation [1–7]. Recently, two-dimensional (2D) layered MoS_2_ has attracted considerable research attention as a promising semiconductor photocatalyst because of its excellent catalytic activity, high chemical stability, eco-friendliness, and abundance in nature [2–4]. In particular, few-layer-thick MoS_2_ nanosheets can be central to exploiting the full potential of 2D MoS_2_ for solar-light PEC reactions because of the feasibility of mass production and appropriate bandgap energy, which is tunable from ~ 1.2 eV for indirect gap in the bulk form to ~ 1.9 eV for direct gap in the monolayer [8–10]. The PEC activity of 2D MoS_2_, which has strong in-plane covalent bonding of S–Mo–S and weak out-of-plane van der Waals interaction between neighboring S–S layers, is significantly hindered by poor charge transport across basal layers through hopping [2–4]. Thus, the ideal architecture configuration comprises 2D MoS_2_ nanosheets that stand vertically on electrode substrates because the highly conductive edges of MoS_2_ provide an efficient pathway for photoexcited carriers and good electronic contact with the substrate. In addition, vertically packed 2D sheets offer higher volume than laid sheets for interacting with incoming photon flux on a unit substrate area. He et al. demonstrated that the edge-on structure of MoS_2_ flakes/TiO_2_ nanowires improves the photocatalytic hydrogen evolution of MoS_2_ [11]. Recently, we reported the enhanced PEC activity of few-layer MoS_2_ nanosheets vertically grown on supporting electrode substrates, such as indium-tin oxide (ITO) and ITO/TiO_2_ nanowires [12, 13]. However, the synthesis and PEC applications of vertically aligned few-layer MoS_2_ nanosheets on graphene have not been reported despite its considerable potential.

The support substrate should be made highly conductive and form an appropriate energy band alignment with MoS_2_ to minimize Ohmic junction losses. Graphene has attracted huge research attention as a promising conducting layer not only because it displays remarkable electron mobility (> 15,000 cm^2^ V^−1^ s^−1^) but also due to its favorable electric contact with MoS_2_. Chang et al. reported the enhanced photocatalytic hydrogen evolution of MoS_2_/graphene as a result of the improved charge transport of graphene [14]. Carraro et al. demonstrated the one-pot aerosol synthesis of MoS_2_ nanoparticles/graphene for enhanced PEC hydrogen production [15]. Biroju et al. also reported that an adequate stacking of 2D MoS_2_ and graphene exhibited a Δ*G*_H_ value that is close to zero, which is ideal for hydrogen evolution reactions [16]. However, most MoS_2_/graphene heterostructures have been synthesized by wet-chemical and mechanical transfer approaches, which are unsuitable for controlled synthesis or vertical stacking of few-layer MoS_2_ nanosheets on graphene electrode substrates [14–18]. Wet-chemical synthesis methods have yielded a wide range of MoS_2_ layer thicknesses and produced the randomly assembled structures of 2D MoS_2_ and graphene [14, 15, 19].

The optical absorption of 2D MoS_2_ can be remarkably enhanced by employing plasmonic metal nanoparticles (NPs), such as Ag or Au. Plasmonic metals improve the optical absorption over the entire solar spectrum as well as broaden and tune the optical absorption behavior, depending on their composition, size, and shape [20, 21]. Ag and Au have gained research interest because of their strong resonance with ultraviolet (UV) and visible light. Moreover, the surface plasmon resonance (SPR) of metal NPs enhances the intensity of the electric field near the metal NPs, thereby significantly increasing the rate of electron–hole (e–h) pair generation [20, 22]. Plasmonic metal NPs also act as dye sensitizers by absorbing resonant photons and injecting high-energy electrons into the nearby semiconductor [20, 23]. Kang et al. reported the effective injection of SPR-excited electrons, i.e., hot electrons, by Au NPs into the conduction band of MoS_2_ by overcoming the Schottky barrier (~ 0.8 eV) of Au/MoS_2_ [24]. Plasmonic effect has been effectively applied to enhance the photocatalytic and PEC performances of various semiconductors, such as TiO_2_, CdS, ZnO, and BiFeO_3_ [25–32]. To maximize the SPR effects and enhance the PEC activity, SPR-induced charge carriers should be efficiently transported to the corresponding electrode/water interfaces. Vertically aligned few-layer MoS_2_ nanosheets on graphene can act as desirable heterostructures to synergistically exploit the SPR effects in terms of energy band diagram and physical nanostructure architecture.

**Fig. 1:**
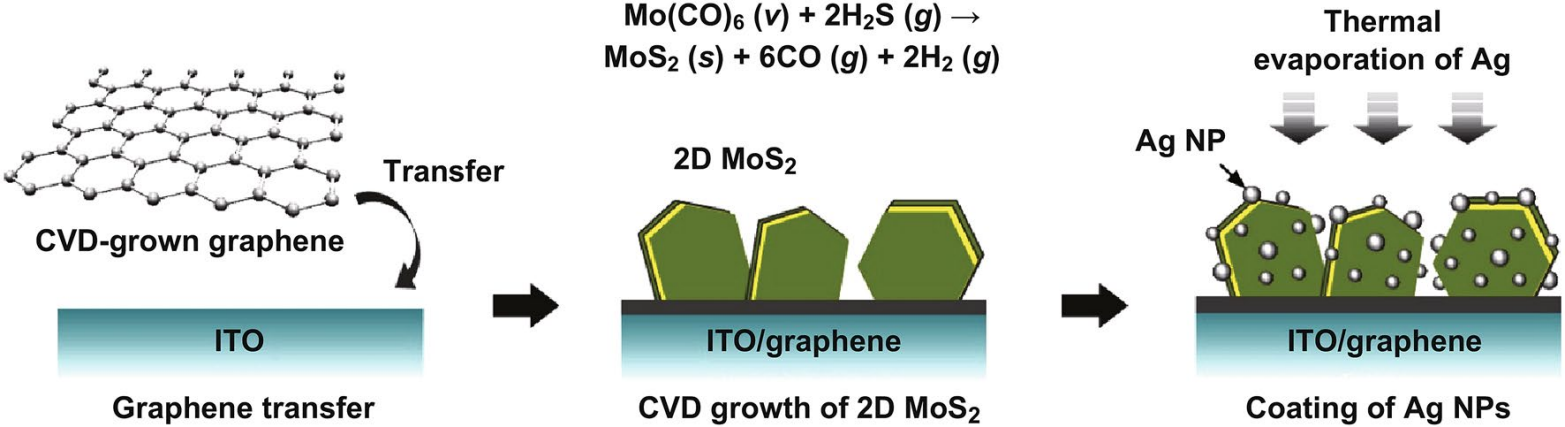
Schematic of preparation of plasmonic Ag-decorated vertically aligned few-layer MoS_2_ nanosheets on graphene

Herein, we report significantly improved PEC efficiency through the synergetic effects of (1) SPR-enhanced optical absorption and photo-generation of charge carriers and (2) efficient separation and transportation of photo-generated e–h pairs through few-layer MoS_2_ sheets that are vertically aligned on graphene. Few-layer MoS_2_ sheets were vertically grown on graphene in a controlled manner at relatively low temperatures (250 °C) through metalorganic chemical vapor deposition (MOCVD) to minimize damage to the graphene. For the SPR effect, Ag NPs were formed on 2D MoS_2_ sheets on graphene through simple thermal evaporation of Ag (Fig. 1). Thermal evaporation is a low-cost and practical method for large-area substrates in comparison with previously reported methods, such as sophisticated metal nanopatterning [21], drop/spin casting of pre-synthesized metal NPs [33], and chemical synthesis [22]. Ag was chosen as plasmonic metal because of its appropriate resonant wavelength range in the UV to near-infrared (IR) band and smaller work function (~ 4.3–4.8 eV) than Au (~ 5.1–5.5 eV) and Pt (~ 5.1–5.9 eV). Thus, Ag/MoS_2_ forms a low Schottky barrier, which is advantageous for the efficient injection of SPR-excited electrons into the conduction band of MoS_2_.

## Experimental

### Preparation of heterostructures of graphene/2D MoS_2_/Ag NPs

Graphene was synthesized on Cu foils (Alfa Aesar) by using inductively coupled plasma (ICP) CVD with CH_4_ and H_2_ gases at 950 °C for 5 min. The ICP power and growth pressure were fixed at 200 W and 1 Torr, respectively. The synthesized graphene on Cu was transferred on an ITO glass substrate (Fig. 1). The CVD growth and transfer procedures of graphene were further described elsewhere [34]. MoS_2_ was directly grown on ITO and ITO/graphene at 250 °C by using MOCVD with Mo(CO)_6_ and H_2_S gas (5 vol% in balance N_2_) as Mo and S precursors, respectively. Mo(CO)_6_ was vaporized at 20 °C and transferred into a quartz reaction tube with Ar gas of 25 standard cubic centimeters per minute (SCCM). The flow rate of H_2_S gas was 75 SCCM. The growth pressure and time were fixed at 1 Torr and 5 min, respectively. Ag NPs were formed on ITO/graphene/MoS_2_ through thermal evaporation of Ag at room temperature. The size and coverage of Ag NPs on few-layer MoS_2_ were controlled by various nominal Ag deposition thicknesses of 2, 4, and 8 nm. The Ag contents per electrode area were estimated to be 2.1, 4.2, and 8.4 μg cm^−2^ for 2, 4, and 8 nm of Ag, respectively.

### Characterization

The morphology of the samples was investigated via scanning electron microscopy (SEM; Hitachi S-4800) and transmission electron microscopy (TEM; Tecnai G^2^ F30 S-Twin). The structural properties of MoS_2_ were characterized by TEM and micro-Raman spectroscopy by using an excitation band of 532 nm and a charge-coupled device detector. The chemical states and composition of the samples were characterized by X-ray photoelectron spectroscopy (XPS; Thermo Fisher K-Alpha+). Optical properties were evaluated by UV–visible (UV–Vis; Scinco S-3100) and photoluminescence (PL) spectroscopy (excitation at 532 nm). Photoexcited carrier behavior was investigated by time-resolved PL (TRPL) measurements. The samples were excited using a 467 nm pulsed laser, and the transient signal was recorded using a time-correlated single-photon counting spectrometer (Horiba Fluorolog 3). The energy level of MoS_2_ was evaluated via UV photoelectron spectroscopy (UPS; Thermo scientific, K-alpha^+^).

### Photoelectrochemical Measurement

PEC cells were fabricated on 1 × 2 cm^2^ ITO glass substrates. The working area of the PEC cells was fixed at 0.5 × 0.5 cm^2^ by using non-conductive epoxy to cover the undesired areas. PEC characterization was performed using a three-electrode system and an electrochemical analyzer (potentiostat/galvanostat 263A). A Pt plate and KCl-saturated calomel (Hg/Hg_2_Cl_2_) were used as counter and reference electrodes, respectively. The electrolyte solutions were prepared with 0.3 M KH_2_PO_4_ + 0.3 M KOH and 0.5 M Na_2_SO_3_ + 0.5 M Na_2_SO_4_. The light source was simulated AM 1.5G irradiation of 100 Mw cm^−2^ delivered by a 150 W Xe arc lamp. The current density–voltage characteristics were recorded using a source meter (Keithley 2400). Electrochemical impedance spectroscopy (EIS) measurement was performed under constant light illumination (100 mW cm^−2^) at a bias of 0.6 V with varying AC frequencies from 100 kHz to 100 mHz. The incident monochromatic photon-to-current conversion efficiency (IPCE) of the electrode structure was measured using a grating monochromator in the excitation wavelength range of 300–800 nm. The hydrogen gas products were analyzed using a YL 6500 gas chromatograph (Young In Chromass Co., Ltd.) equipped with a flame ionization detector and a thermal conductivity detector. A gas volume of 0.5 mL was injected into columns of 40/60 Carboxen-1000 for GC analysis.

## Results and Discussion

### Microstructure of AgNP-Decorated MoS_2_ Nanosheets on Graphene

**Fig. 2:**
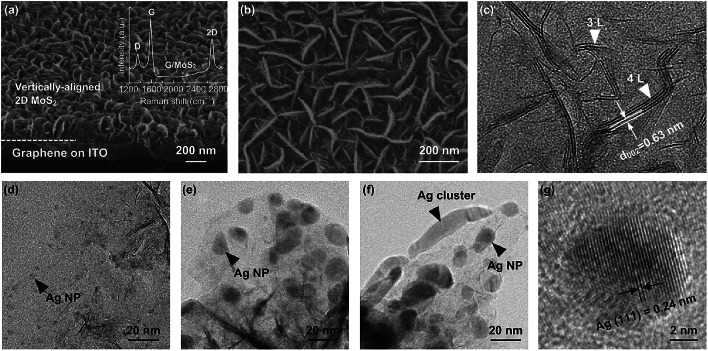
**a** Tilted-view SEM, **b** planar-view SEM, and **c** planar-view TEM images of vertically aligned MoS_2_ nanosheets on graphene (G/MoS_2_). The inset in **a** is the Raman spectrum of G/MoS_2_. TEM images of Ag-decorated vertically aligned few-layer MoS_2_ nanosheets on graphene: **d** G/MoS_2_/Ag-2, **e** G/MoS_2_/Ag-4, and **f** G/MoS_2_/Ag-8. **g** High-resolution lattice TEM image of an Ag NP in G/MoS_2_/Ag-4

MoS_2_ nanosheets with a height of ~ 200 nm and length of ~ 150–250 nm were vertically aligned and densely packed on the ITO/graphene substrate (hereinafter referred to as G/MoS_2_, Fig. 2a, b). Owing to the low-temperature growth at 250 °C, the graphene layer remained after the MOCVD growth of MoS_2_, as confirmed by the presence of the characteristic G and 2D band peaks in the Raman spectrum (inset of Fig. 2a, Fig. S1a, b). The pristine CVD-grown graphene layer exhibited a low-intensity ratio of D to G band peaks (> 0.15) and an excellent light transmittance of 96.8% at 550 nm (Fig. S1c), corresponding to approximately one and a half layers of high-quality graphene [34]. The structure of few-layer MoS_2_ was investigated using TEM and Raman spectroscopy. The planar-view TEM image of G/MoS_2_ clearly showed the layered structure of MoS_2_ sheets with edges on graphene (Fig. 2c). The sheets comprised 1–5 layers with an interlayer spacing of 0.63 nm, corresponding to the semiconducting 2H MoS_2_. The TEM results are consistent with the Raman spectrum of G/MoS_2_ (Fig. S2a). The $E^{1}_{{2{\text{g}}}}$ and *A*_1g_ modes can be attributed to the in-plane vibration of Mo and S atoms and the out-of-plane vibration of S atoms, respectively. The positions and relative frequency difference (RFD) of $E^{1}_{{2{\text{g}}}}$ and the *A*_1g_ peaks are strongly correlated with the number of MoS_2_ layers [12, 35, 36]. For G/MoS_2_, the RFD value (22.3 cm^−1^) of $E^{1}_{{2{\text{g}}}}$ (385.0 cm^−1^) and *A*_1g_ peaks (407.3 cm^−1^) corresponds to a few layers of MoS_2_. The MoS_2_ sheets grown on ITO (hereinafter referred to as ITO/MoS_2_) showed similar size and morphology as the counterpart sample, namely G/MoS_2_ (Fig. S2b). The RED value of $E^{1}_{{2{\text{g}}}}$ and *A*_1g_ peaks of ITO/MoS_2_ was also similar to that of G/MoS_2_ (Fig. S2a).

**Fig. 3:**
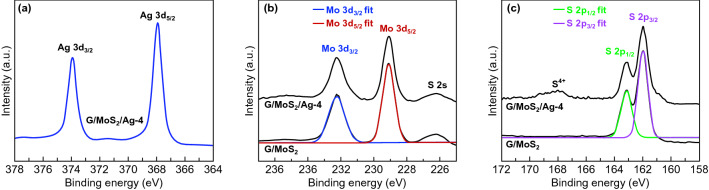
XPS spectra of **a** Ag 3*d*, **b** Mo 3*d*, and **c** S 2*p* core levels in G/MoS_2_ and G/MoS_2_/Ag-4

Figure 2d–f shows the TEM images of Ag-decorated G/MoS_2_ with nominal Ag thicknesses of 2, 4, and 8 nm, respectively (referred to as G/MoS_2_/Ag-2, G/MoS_2_/Ag-4, and G/MoS_2_/Ag-8, respectively). The size of the Ag NPs on MoS_2_ was successfully manipulated by varying the nominal deposition thickness of Ag through thermal evaporation. For G/MoS_2_/Ag-2, Ag NPs with a size of ~ 3–5 nm were formed on the MoS_2_ nanosheet surface (Fig. S3). The NP size increased to ~ 10–20 nm for G/MoS_2_/Ag-4. By increasing the nominal deposition thickness to 8 nm, the size of Ag NPs increased to ~ 20–40 nm. In addition, large Ag clusters of ~ 60–100 nm were partially formed. The high-resolution TEM lattice image revealed that the NPs were metallic Ag (Fig. 2g). The metallic Ag was also confirmed by XPS. Figure 3a shows two strong peaks in the XPS spectrum of G/MoS_2_/Ag-4 at 373.9 and 367.9 eV, which can be attributed to the Ag 3*d*_3/2_ and Ag 3*d*_5/2_ orbitals of metallic Ag, respectively [37].

MoS_2_ presents two common structure polymorphs, namely semiconducting 2H and metallic 1T phases, which can be converted from each other by surface treatments, such as Ar-plasma bombardment and metal deposition [2, 38, 39]. As shown in Fig. 3b, the XPS spectra of the Mo 3*d* core level were deconvoluted into only two peaks at 229.1 and 232.2 eV, which can be attributed to the Mo^4+^ 3*d*_5/2_ and Mo^4+^ 3*d*_3/2_ components of the 2H phase of MoS_2_, respectively [2, 38, 39]. Pristine MoS_2_ (G/MoS_2_) and Ag NP-decorated MoS_2_ (G/MoS_2_/Ag-4) exhibited nearly identical XPS spectra at the Mo 3*d* core level, indicating that the single phase of semiconducting 2H MoS_2_ remained stable after Ag NP decoration. This structural stability is highly advantageous for 2D MoS_2_ in semiconducting photoelectrode applications. Figure 3c shows the XPS spectra of the S 2*p* core level of MoS_2_. The spectra were deconvoluted into two peaks at 163.2 and 162.0 eV, corresponding to the S 2*p*_1/2_ and S 2*p*_3/2_ orbital of divalent sulfur, respectively [38]. The ratios of the S 2*p*_1/2_ and S 2*p*_3/2_ peaks of G/MoS_2_ and G/MoS_2_/Ag-4 were almost identical, suggesting a single phase of 2H MoS_2_ for both samples. The 2H phase of both samples was also confirmed by TEM (Fig. 2c). G/MoS_2_/Ag-4 exhibited a small broad bump near 168 eV, which can be attributed to S^4+^ due to Ag sulfurization. However, the peak was removed by slight Ar-plasma surface etching, indicating the ultrathin layer of silver sulfide. The XPS result implies that the interface of MoS_2_/Ag is likely to be an alloy interface.

### Effect of Graphene on the PEC Activity of MoS_2_ Nanosheets

**Fig. 4:**
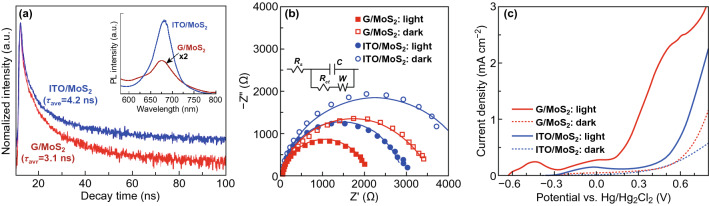
**a** TRPL results of ITO/MoS_2_ and G/MoS_2_. The inset shows the corresponding PL spectra. **b** Nyquist plots of ITO/MoS_2_ and G/MoS_2_ in the dark and under illumination. The inset shows the equivalent Randles circuit. **c** Photo- and dark current densities versus the potential curves of PEC cells with working electrodes of ITO/MoS_2_ and G/MoS_2_

The G/MoS_2_ and ITO/MoS_2_ samples exhibited a PL peak at 676 nm (inset of Fig. 4a), which is consistent with the energy of exciton A. Hence, the dominant electronic transition was the direct bandgap transitions at the *Κ* point [12]. Notably, G/MoS_2_ achieved significantly lower PL efficiencies than ITO/MoS_2_. The PL quenching efficiency indicated that the graphene layer played a crucial role in reducing the e–h recombination in MoS_2_. TRPL spectroscopy study was conducted to further understand the dynamic behavior of photo-generated carriers (Fig. 4a). The average carrier lifetimes were extracted using the PL decay kinetics fitted by a bi-exponential decay profile [40]. G/MoS_2_ exhibited a shorter carrier lifetime of 3.1 ns than ITO/MoS_2_ (4.2 ns). The reduced carrier lifetime can be attributed to the benefits of graphene/MoS_2_ heterojunction for efficient separation and transportation of photo-generated carriers to the semiconductor/liquid interface [18].

EIS study was conducted to further understand the charge transport property. Figure 4b shows the Nyquist plots of EIS in the dark and under illumination. G/MoS_2_ exhibited smaller EIS semicircles than ITO/MoS_2_, whose radius mirrors the charge transfer resistance (*R*_ct_). The Nyquist plots were fitted using a simplified Randles circuit (inset of Fig. 4b), consisting of *R*_ct_, solution resistance (*R*_s_), constant phase element (*Q*), and diffusion of species in electrolyte solution represented by Warburg impedance (*W*). The *R*_ct_ values are listed in Supporting Information (Table S1). G/MoS_2_ had *R*_ct_ values of 3264 and 1959 Ω, whereas ITO/MoS_2_ exhibited *R*_ct_ values of 4236 and 2766 Ω in the dark and under illumination, respectively. Moreover, the *R*_ct_ (dark)-to-*R*_ct_ (photo) ratio (1.67) of G/MoS_2_ was greater than that of ITO/MoS_2_ (1.53), suggesting that the photo-generated e–h pairs were efficiently separated and transported through the graphene/MoS_2_ heterojunction.

Considering the benefits of graphene/MoS_2_ heterojunction, G/MoS_2_ exhibited significantly higher PEC activity through the measured potential range than ITO/MoS_2_ (Fig. 4c). G/MoS_2_ yielded approximately nine times higher photocurrent density (1.72 mA cm^−2^) at 0.4 V (at which the photo-to-dark current ratio (*I*_ph_/*I*_dark_) was at maximum) than ITO/MoS_2_ (0.19 mA cm^−2^). The maximum *I*_ph_/*I*_dark_ value of G/MoS_2_ was approximately 16 at 0.4 V, whereas that of ITO/MoS_2_ was approximately 4 at 0.8 V. The water oxidation onset potential (~ 0.13 V), which is generally defined by the potential at the intersection of the dark current and the tangent at the maximum slope of the photocurrent, of G/MoS_2_ had a cathodic shift of ~ 0.41 V with respect to that (~ 0.54 V) of ITO/MoS_2_. The rapidly increasing photocurrent density of the samples above 0.6 V resulted from the considerably high dark current, which can be attributed to the electrocatalysis and electro-corrosion of MoS_2_, in which active S atoms can react with redox species in the solution [12, 14].

### Effect of Plasmonic Ag NPs on the PEC Activity of Graphene/MoS_2_ Nanosheets

**Fig. 5:**
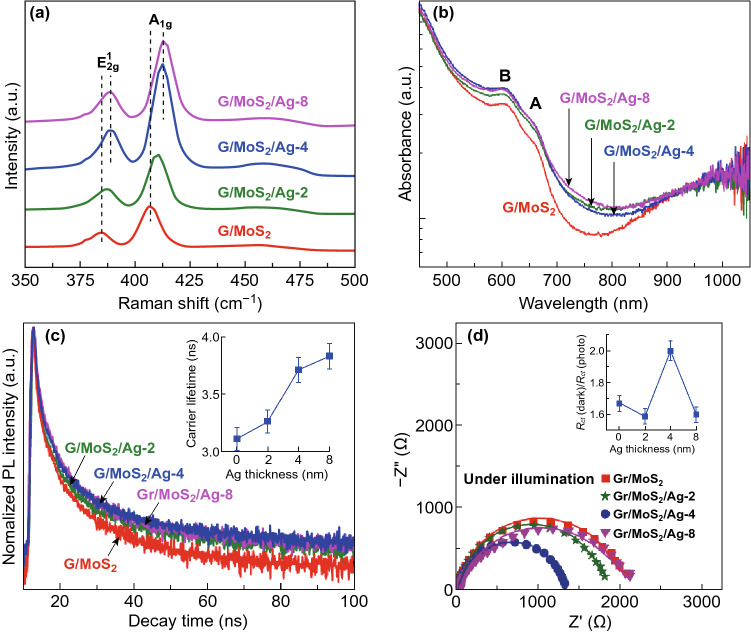
**a** Raman spectra, **b** UV–Vis absorption, **c** TRPL results, and **d** Nyquist plots of G/MoS_2_, G/MoS_2_/Ag-2, G/MoS_2_/Ag-4, and G/MoS_2_/Ag-8. The inset in **c** shows the carrier lifetimes based on the corresponding TRPL measurements. The inset in **d** shows the *R*_ct_ (dark)/*R*_ct_ (photo) values of G/MoS_2_, G/MoS_2_/Ag-2, G/MoS_2_/Ag-4, and G/MoS_2_/Ag-8

Raman spectroscopy characterization was performed to investigate the interaction of Ag NPs with MoS_2_. The characteristic $E^{1}_{{2{\text{g}}}}$ and *A*_1g_ modes of the Ag-decorated samples were redshifted with respect to those of G/MoS_2_-250 because of the stiffening of $E^{1}_{{2{\text{g}}}}$ and *A*_1g_ vibrations (Fig. 5a). The stiffened lateral vibration between Mo and S atoms through $E^{1}_{{2{\text{g}}}}$ mode resulted from the p-doping effect of Ag NPs in MoS_2_ [38]. MoS_2_/Ag was very likely to form a Schottky junction because of electron transfer from MoS_2_ to Ag NPs, as shown in Fig. S4. The stiffened vertical vibration of S atoms through *A*_1g_ mode was also attributed to the interaction between the Ag NPs and MoS_2_ [23]. Figure 5b shows the UV–Vis absorption spectra of G/MoS_2_ and the Ag-decorated MoS_2_ samples. All samples showed two prominent absorption peaks at approximately 607 and 663 nm. The two peaks, known as excitons B and A, respectively, can be attributed to the direct excitonic transitions at the *K* point of the MoS_2_ Brillouin zone [12, 41]. In comparison with G/MoS_2_, the Ag-decorated MoS_2_ samples exhibited stronger absorption intensity, especially for red light and near-IR regions. Additionally, the absorption edges (~ 800 nm) of Ag-decorated MoS_2_ samples were redshifted with respect to that (~ 750 nm) of G/MoS_2_. The enhanced visible light and broadened absorption near the IR region can be attributed to the strong coupling between the excitons and surface plasmons of Ag NPs [20, 21]. The PL spectra also exhibited redshifting behavior with increasing Ag NP sizes (Fig. S5). G/MoS_2_ showed a PL peak position of 676 nm, corresponding to the energy of the exciton A, representing the direct bandgap transitions at the *Κ* point of 2D MoS_2_. With increasing Ag NP sizes, the PL peak was gradually redshifted, reaching 688 nm for G/MoS_2_/Ag-8.

TRPL study was conducted to investigate the plasmonic effect of Ag NPs on the dynamic carrier behavior. G/MoS_2_ exhibited the shortest carrier lifetime of 3.1 ns, whereas G/MoS_2_/Ag-4 and G/MoS_2_/Ag-8 yielded longer carrier lifetimes of ~ 3.8 ns (Fig. 5c). The carrier lifetime increased with increasing nominal Ag deposition thicknesses. The long carrier lifetimes of Ag-decorated MoS_2_ samples can be attributed to suppressed e–h recombination by filling the trapping sites of MoS_2_ by plasmon-excited electrons [23]. Ag-decorated MoS_2_ samples also exhibited smaller EIS semicircles than G/MoS_2_ (Fig. 5d, Fig. S5). The Nyquist plots of G/MoS_2_/Ag-4 yielded the lowest *R*_ct_ in the dark (~ 2572 Ω) and under illumination (~ 1284 Ω), suggesting the effective assistance of the carrier transfer through the Ag NPs on the MoS_2_ surface. Moreover, G/MoS_2_/Ag-4 yielded the highest *R*_ct_ (dark)/*R*_ct_ (photo) of 2.00 (inset of Fig. 5d). The increased *R*_ct_ (dark)/*R*_ct_ (photo) can result from the SPR-enhanced photo-generation and transfer rate of the charge carriers. In addition, the heterojunction of MoS_2_/Ag can play a significant role in improving the charge separation and transfer rate [42, 43].

**Fig. 6:**
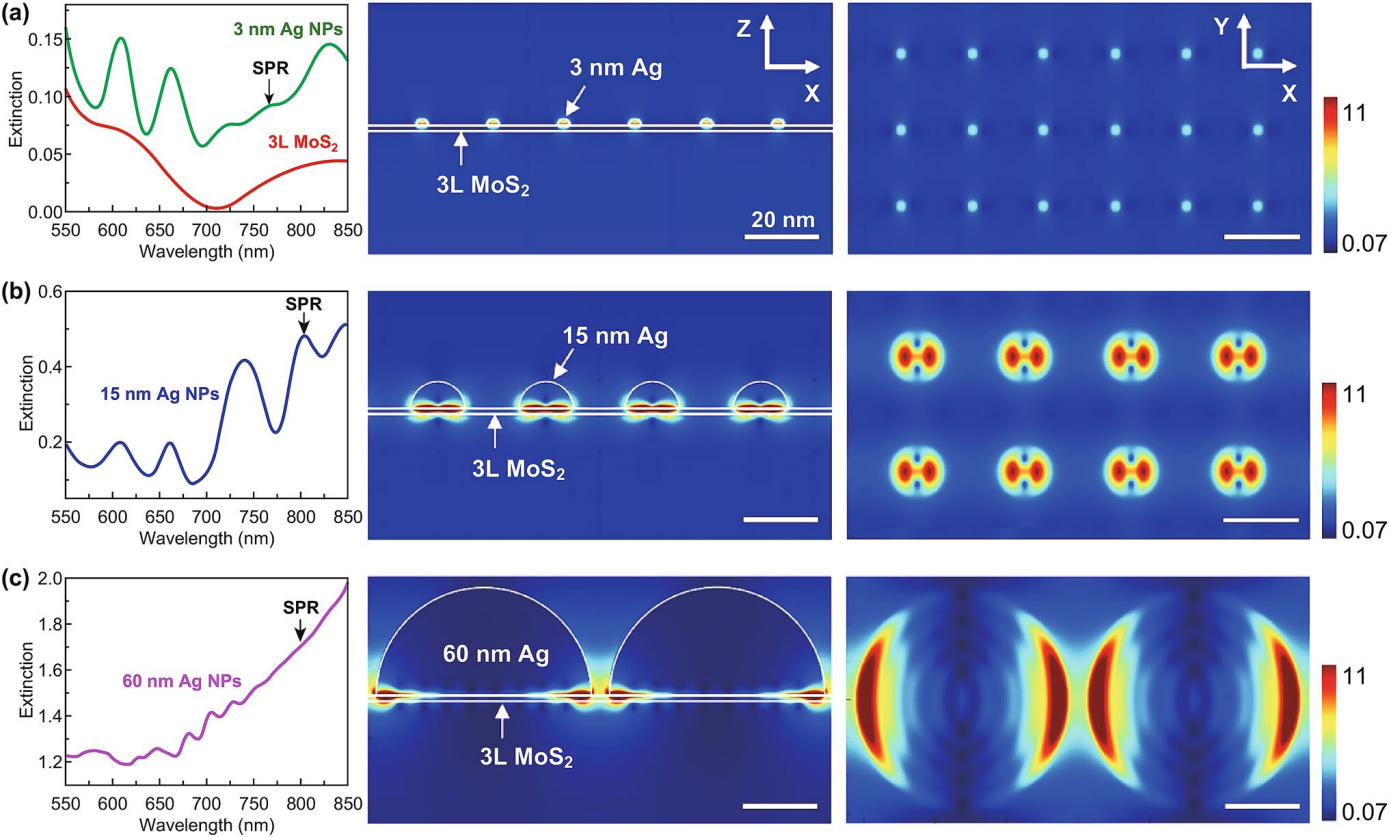
Simulated UV–Vis absorption spectra and electric field distribution contour plots at *λ*_SPR_ for **a** AgNP-3, **b** AgNP-15, and **c** AgNP-60

To validate the experimental findings, finite-difference time-domain (FDTD) simulations of the interaction of materials with the incident electromagnetic radiation were performed based on Maxwell’s equations. Ag hemispheres in an open-air environment under the illumination of a plane-wave source were considered (Fig. S7). A 2D periodic orientation of the NPs on a three-layer-thick MoS_2_ substrate was assumed including the following: (1) 3-nm-diameter NPs with a pitch of 20 nm (referred to as AgNP-3), (2) 15-nm-diameter NPs with a pitch of 30 nm (referred to as AgNP-15), and (3) 60-nm-diameter NPs with a pitch of 65 nm (referred to as AgNP-60). The electric field vector of the source oscillated along the *x*-axis, while the propagation vector is along the *z*-axis. Figure 6 shows the simulated UV–Vis absorption spectra and electric field distribution contour plots at the wavelength of SPR (*λ*_SPR_) for AgNP-3, AgNP-15, and AgNP-60. The simulated UV–Vis absorption spectrum of pristine MoS_2_ substrate was in good agreement with the corresponding experimental result (Fig. 6a). The light absorption was significantly enhanced in the visible light and IR regions by Ag NP decoration. Notably, the SPR peaks appeared above the absorption edge of pristine MoS_2_ (~ 700 nm), consistent with the experimentally observed broadened absorption near the IR region. AgNP-15 exhibited the strongest SPR effect, resulting in the highly amplified local electric field intensity in MoS_2_ (Fig. 6b). The SPR-enhanced electric field can increase the rate of e–h pair generation by a few orders of magnitude [20]. The 15 nm Ag NPs induced an SPR-enhanced electric field in the entire interface region of MoS_2_/Ag NP. By contrast, the 60 nm Ag NPs showed SPR effect only along the edge of NPs (Fig. 6c), indicating that a significant amount of light was extinguished by big Ag clusters (~ 60–100 nm) of G/MoS_2_/Ag-8 without photo-generation of e–h pairs in MoS_2_. The excessive surface coverage of metal NPs can also hinder contact of the electrochemical active surface with the electrolyte solution, resulting in deteriorated PEC activity [44, 45]. In addition, the surface plasmons of small Ag NPs (< 30 nm) undergo decay because of the formation of energetic charge carriers, but those of large Ag NPs (> 50 nm) undergo decay through the radiative scattering of resonant photons [37, 46]. Thus, the Ag NPs of G/MoS_2_/Ag-4 efficiently injected SPR-excited electrons into the conduction band of MoS_2_, resulting in the largest *R*_ct_ (dark)/*R*_ct_ (photo). Meanwhile, the SPR effect of G/MoS_2_/Ag-2 was relatively weak because of its low surface coverage of tiny Ag NPs of less than 10 nm (Fig. 6a).

**Fig. 7:**
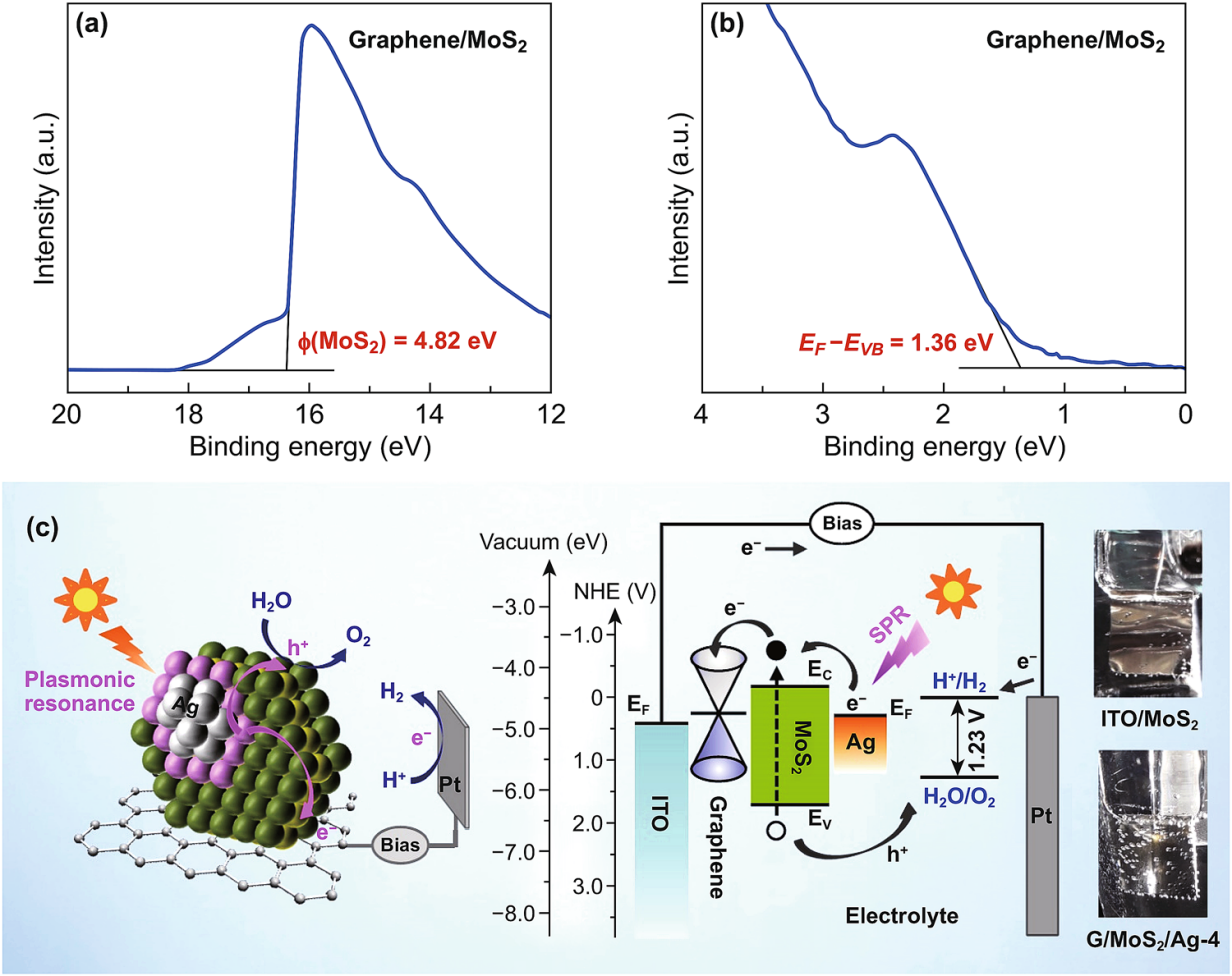
**a** UPS secondary electron cutoff and **b** valence spectra of G/MoS_2_. **c** PEC water-splitting working principle of plasmonic Ag-decorated vertically aligned few-layer MoS_2_ nanosheets on graphene. The photographs in **c** show gas bubbling on the dark cathodes (Pt) for ITO/MoS_2_ and G/MoS_2_/Ag-4 during PEC measurement

To gain insight into the carrier transport property across the heterojunction of graphene/MoS_2_, its electronic structure was studied by UPS. The work function of MoS_2_ (4.82 ± 0.15 eV) was determined based on the difference between the photon energy of excited radiation (21.2 eV) and the spectrum width which is measured from the valence band and secondary edges (16.38 eV, Fig. 7a). The energy difference between the Fermi energy and valence band edge (*E*_F_–*E*_VB_) was 1.36 eV (Fig. 7b). Considering the bandgap energy of ~ 1.88 eV for MoS_2_ based on the UV–Vis absorption and PL spectra, the electron affinity of MoS_2_ was approximately 4.33 eV, which is consistent with the previously reported values (~ 4.3 eV) [47]. This electronic structure suggests the n-type behavior of MoS_2_, working as a photoanode. As shown in Fig. 7c, the Fermi level (~ 4.6–4.8 eV of work function) of pristine few-layer graphene [48] was appropriately located between the Fermi level of the ITO and the conduction band edge of MoS_2_ for efficient extraction of electrons to the cathode.

**Fig. 8:**
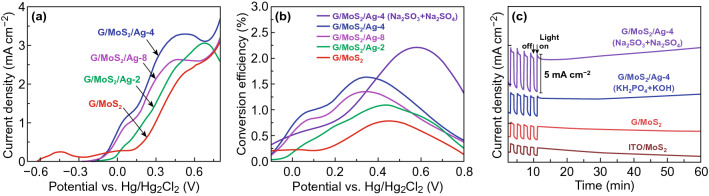
**a** Photocurrent density–potential curves of PEC cells with various working electrodes (G/MoS_2_, G/MoS_2_/Ag-2, G/MoS_2_/Ag-4, and G/MoS_2_/Ag-8) in 0.3 M KH_2_PO_4_ + 0.3 M KOH solution. **b** Photoconversion efficiencies and **c** photocurrent–time plots for G/MoS_2_, G/MoS_2_/Ag-2, G/MoS_2_/Ag-4, and G/MoS_2_/Ag-8 in 0.3 M KH_2_PO_4_ + 0.3 M KOH solution and G/MoS_2_/Ag-4 in 0.5 M Na_2_SO_3_ + 0.5 M Na_2_SO_4_ solution

Figure 8a shows the linear sweep voltammograms of G/MoS_2_, G/MoS_2_/Ag-2, G/MoS_2_/Ag-4, and G/MoS_2_/Ag-8 under illumination. The Ag-decorated MoS_2_ samples exhibited significantly higher PEC activities than G/MoS_2_, whereas the dark currents were almost identical (Fig. S8). Subsequently, G/MoS_2_/Ag-4 yielded 2.5 times higher *I*_ph_/*I*_dark_ value than G/MoS_2_. In addition, the Ag-decorated MoS_2_ samples showed a significant cathodic shift of onset potential up to 0.2 V with respect to that of G/MoS_2_. The Ag-decorated MoS_2_ samples also did not exhibit any anodic peaks before the onset potential, whereas G/MoS_2_ yielded minor but noticeable peaks. The anodic peaks can be originated from surface states of 2D MoS_2_ nanosheets, which can be filled and deactivated by plasmon-excited electrons [23]. Passivating surface states also caused the significant cathodic shift of onset potential [49] and prolonged the carrier lifetimes of the Ag-decorated MoS_2_ samples (Fig. 5c). Photoconversion efficiency (*η*) was estimated using the following equation to further quantify PEC performance [50]:

1 $$\eta = J\left( {E^{o} {-}V_{\text{app}} } \right)/P_{\text{light}}$$

where *J* is the photocurrent density (mA cm^−2^) at the applied potential, *E*^*o*^ is the standard reversible potential (1.23 V), *V*_app_ is applied potential, and *P*_light_ is the power density of illumination. The photoconversion efficiencies of Ag-decorated MoS_2_ samples were significantly higher than that (0.8% at − 0.45 V) of G/MoS_2_ (Fig. 8b). Among the samples, G/MoS_2_/Ag-4 exhibited the highest photoconversion efficiency of 1.6% at − 0.35 V. The photoconversion efficiency of G/MoS_2_/Ag-4 further increased to 2.2% at − 0.58 V in 0.5 M Na_2_SO_3_ + 0.5 M Na_2_SO_4_. The photoconversion efficiency G/MoS_2_/Ag-4 was comparable with those of previously reported photoanodes, such as Au-decorated MoS_2_ flakes on carbon fiber cloth (1.27%) [51], MoS_2_ nanosheets on TiO_2_ nanorods (0.81%) [52], Ag-embedded MoS_2_/BiVO_4_ heterojunctions (2.67%) [53], and MoS_2_ nanosheets on polydopamine-modified TiO_2_ nanotubes (1.56%) [54]. Moreover, the photocurrents of G/MoS_2_ and G/MoS_2_/Ag-4 did not change significantly after 1 h of illumination, whereas the photocurrent of ITO/MoS_2_ decreased continuously (Fig. 8c). The photocurrents of G/MoS_2_ and G/MoS_2_/Ag-4 decayed initially but saturated shortly above 300 s. The decayed photocurrent was attributed to the recombination of the photo-generated holes with electrons [55]. The photocurrent became stable as the transfer and generation of photo-generated e–h pairs reached equilibrium. The stable photocurrent suggests the effective separation and transfer of the photo-generated e–h pairs in the heterojunction of graphene/MoS_2_ nanosheets. To examine the PEC stability of Ag-decorated MoS_2_, the time-dependent PEC measurement of the G/MoS_2_/Ag-4 was repeated after a month. The time-dependent behavior did not change significantly excepting for slightly reduced photocurrents (Fig. S9). After PEC measurement for 1 h, the MoS_2_ nanosheets on ITO (ITO/MoS_2_) were significantly damaged, whereas G/MoS_2_ and G/MoS_2_/Ag-4 showed slight morphological changes (Fig. S10). We recently reported that such morphological changes in MoS_2_ nanosheets were due to the decomposition of MoS_2_, mainly the loss of S elements [12].

**Fig. 9:**
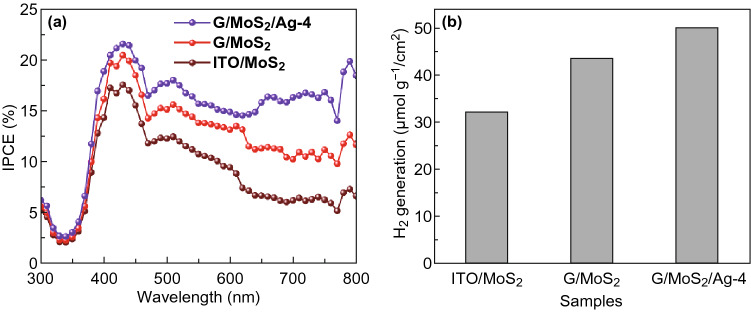
**a** IPCE plots and **b** hydrogen evolution amounts for 15 min of PEC cells with various working electrodes (ITO/MoS_2_, G/MoS_2_, and G/MoS_2_/Ag-4) in 0.5 M Na_2_SO_3_ + 0.5 M Na_2_SO_4_ solution

IPCE and H_2_ evolution studies were carried out to gain insights into the enhanced PEC performance by G/MoS_2_ heterojunction and SPR effects. Figure 9a shows the IPCE plots of various working electrodes (MoS_2_, G/MoS_2_, and G/MoS_2_/Ag-4). G/MoS_2_/Ag-4 exhibited significantly improved photoconversion efficiencies over the overall incident-light waveband, suggesting fortified carrier photo-generation and transfer dynamics. Furthermore, a significant IPCE enhancement was observed at the ~ 650–750 nm region, which was associated with the SPR effect of Ag NPs. Hydrogen evolution from dark cathode (Pt) was measured at 0.6 V versus Hg/Hg_2_Cl_2_ by using a three-electrode configuration during 15 min. The amount of H_2_ produced was significantly increased by G/MoS_2_ heterojunction and SPR effects, suggesting that the photocurrent was attributed to the water-splitting reaction. In addition, the Faradaic efficiency of G/MoS_2_/Ag-4 was estimated to be approximately 90.4%, implying that few side reactions occurred during water splitting [56].

## Conclusion

Plasmonic Ag-decorated vertically aligned few-layer MoS_2_ nanosheets were prepared on graphene in a practical manner through MOCVD of MoS_2_ and thermal evaporation of Ag. G/MoS_2_ showed up to four times higher *I*_ph_/*I*_dark_ than ITO/MoS_2_ because of the efficient separation and transportation of the photo-generated carriers by the graphene/2D MoS_2_ heterojunction. The PEC activity of G/MoS_2_ was further enhanced by plasmonic Ag NP decoration. G/MoS_2_/Ag-4 yielded 10 times higher *I*_ph_/*I*_dark_ value than ITO/MoS_2_. The maximum photoconversion efficiency of G/MoS_2_/Ag-4 was 2.2% at − 0.58 V. The significantly improved PEC performance was attributed to the synergetic effects of SPR and graphene/2D MoS_2_ heterojunction. Plasmonic Ag NPs enhanced visible-light and near-IR absorption of 2D MoS_2_, resulting in significantly increased the photo-generation rate of e–h pairs. Subsequently, the e–h pairs were efficiently separated and transported to catalytic surfaces across the favorable graphene/2D MoS_2_ heterojunction and along the highly conductive edges of the vertically aligned 2D MoS_2_, thereby significantly enhancing the PEC activity. This study offers a practical large-scale approach that combines the potential of SPR and graphene/2D MoS_2_ heterojunction effects for efficient PEC applications.

## Electronic supplementary material

Below is the link to the electronic supplementary material.

Supplementary material 1 (PDF 798 kb)

## References

[1] Walter MG, Warren EL, McKone JR, Boettcher SW, Mi Q, Santori EA, Lewis NS (2010). Solar water splitting cells. Chem. Rev..

[2] Ding Q, Song B, Xu P, Jin S (2016). Efficient electrocatalytic and photoelectrochemical hydrogen generation using MoS_2_ and related compounds. Chem.

[3] Han B, Hu YH (2016). MoS_2_ as a co-catalyst for photocatalytic hydrogen production from water. Energy Sci. Eng..

[4] Chen B, Meng Y, Sha J, Zhong C, Hu W, Zhao N (2018). Preparation of MoS_2_/TiO_2_ based nanocomposites for photocatalysis and rechargeable batteries: progress, challenges, and perspective. Nanoscale.

[5] Chiu YH, Chang TFM, Chen CY, Sone M, Hsu YJ (2019). Mechanistic insights into photodegradation of organic dyes using heterostructure photocatalysts. Catalysts.

[6] Chiu YH, Lai TH, Kuo MY, Hsieh PY, Hsu YJ (2019). Photoelectrochemical cells for solar hydrogen production: challenges and opportunities. APL Mater..

[7] Fang MJ, Tsao CW, Hsu YJ (2020). Semiconductor nanoheterostructures for photoconversion applications. J. Phys. D Appl. Phys..

[8] Li H, Zhang Q, Yap CCR, Tay BK, Edwin THT, Olivier A, Baillargeat D (2012). From bulk to monolayer MoS_2_: evolution of raman scattering. Adv. Funct. Mater..

[9] Mak KF, Lee C, Hone J, Shan J, Heinz TF (2010). Atomically thin MoS_2_: a new direct-gap semiconductor. Phys. Rev. Lett..

[10] Lee C, Yan H, Brus LE, Heinz TF, Hone J, Ryu S (2010). Anomalous lattice vibrations of single- and few-layer MoS_2_. ACS Nano.

[11] He H, Lin J, Fu W, Wang X, Wang H (2016). MoS_2_/TiO_2_ edge-on heterostructure for efficient photocatalytic hydrogen evolution. Adv. Energy Mater..

[12] Trung TN, Seo DB, Quang ND, Kim D, Kim ET (2018). Enhanced photoelectrochemical activity in the heterostructure of vertically aligned few-layer MoS_2_ flakes on ZnO. Electrochim. Acta.

[13] Seo DB, Kim S, Trung TN, Kim D, Kim ET (2019). Conformal growth of few-layer MoS_2_ flakes on closely-packed TiO_2_ nanowires and their enhanced photoelectrochemical reactivity. J. Alloys Compd..

[14] Chang K, Mei Z, Wang T, Kang Q, Ouyang S, Ye J (2014). MoS_2_/graphene cocatalyst for efficient photocatalytic H_2_ evolution under visible light irradiation. ACS Nano.

[15] Carraro F, Calvillo L, Cattelan M, Favaro M, Righetto M (2015). Fast one-pot synthesis of MoS2/crumpled graphene p–n nanojunctions for enhanced photoelectrochemical hydrogen production. ACS Appl. Mater. Interfaces.

[16] Biroju RK, Das D, Sharma R, Pal S, Mawlong LPL (2017). Hydrogen evolution reaction activity of graphene–MoS_2_ van der waals heterostructures. ACS Energy Lett..

[17] Huang Z, Han W, Tang H, Ren L, Chander DS, Qi X, Zhang H (2015). Photoelectrochemical-type sunlight photodetector based on MoS_2_/graphene heterostructure. 2D Mater..

[18] Yu X, Du R, Li B, Zhanga Y, Liu H, Qu J, An X (2016). Biomolecule-assisted self-assembly of CdS/MoS_2_/graphene hollow spheres as high-efficiency photocatalysts for hydrogen evolution without noble metals. Appl. Catal. B.

[19] Zhou W, Zhou K, Hou D, Liu X, Li G (2014). Three-dimensional hierarchical frameworks based on MoS_2_ nanosheets self-assembled on graphene oxide for efficient electrocatalytic hydrogen evolution. ACS Appl. Mater. Interfaces..

[20] Linic S, Christopher P, Ingram DB (2011). Plasmonic-metal nanostructures for efficient conversion of solar to chemical energy. Nat. Mater..

[21] Zu S, Li B, Gong Y, Li Z, Ajayan PM, Fang Z (2016). Active control of plasmon–exciton coupling in MoS_2_–Ag hybrid nanostructures. Adv. Opt. Mater..

[22] Ali A, Mangrio FA, Chen X, Dai Y, Chen K (2019). Ultrathin MoS_2_ nanosheets for high-performance photoelectrochemical applications via plasmonic coupling with Au nanocrystals. Nanoscale.

[23] Shi Y, Wang J, Wang C, Zhai T-T, Bao W-J (2015). Hot electron of Au nanorods activates the electrocatalysis of hydrogen evolution on MoS_2_ nanosheets. J. Am. Chem. Soc..

[24] Kang Y, Najmaei S, Liu Z, Bao Y, Wang Y (2014). Plasmonic hot electron induced structural phase transition in a MoS_2_ monolayer. Adv. Mater..

[25] Chiu YH, Naghadeh SB, Lindley SA, Lai TH, Kuo MY (2019). Yolk–shell nanostructures as an emerging photocatalyst paradigm for solar hydrogen generation. Nano Energy.

[26] Chiu YH, Chang KD, Hsu YJ (2018). Plasmon-mediated charge dynamics and photoactivity enhancement for Au-decorated ZnO nanocrystals. J. Mater. Chem. A.

[27] Li JM, Cheng HY, Chiua YH, Hsu YJ (2016). ZnO–Au–SnO_2_ Z-scheme photoanodes for remarkable photoelectrochemical water splitting. Nanoscale.

[28] Huang YL, Chang WS, Van CN, Liu HJ, Tsai KA (2016). Tunable photoelectrochemical performance of Au/BiFeO_3_ heterostructure. Nanoscale.

[29] Van CN, Chang WS, Chen JW, Tsai KA, Tzeng WY (2015). Heteroepitaxial approach to explore charge dynamics across Au/BiVO_4_ interface for photoactivity enhancement. Nano Energy.

[30] Chen YC, Liu TC, Hsu YJ (2015). ZnSe·0.5N_2_H_4_ hybrid nanostructures: a promising alternative photocatalyst for solar conversion. ACS Appl. Mater. Interfaces..

[31] Pu YC, Wang G, Chang KD, Ling Y, Lin YK (2013). Au nanostructure-decorated TiO_2_ nanowires exhibiting photoactivity across entire UV–visible region for photoelectrochemical water splitting. Nano Lett..

[32] Chen KH, Pu YC, Chang KD, Liang YF, Liu CM (2012). Ag-nanoparticle-decorated SiO_2_ nanospheres exhibiting remarkable plasmon-mediated photocatalytic properties. J. Phys. Chem. C.

[33] Li YY, Wang JH, Luo ZJ, Chen K, Cheng ZQ (2017). Plasmon-enhanced photoelectrochemical current and hydrogen production of (MoS_2_–TiO_2_)/Au hybrids. Sci. Rep..

[34] Nang LV, Kim ET (2012). Controllable synthesis of high-quality graphene using inductively-coupled plasma chemical vapor deposition. J. Electrochem. Soc..

[35] Jeon J, Jang SK, Jeon SM, Yoo G, Jang YH, Park J-H, Lee S (2015). Layer-controlled CVD growth of large-area two-dimensional MoS_2_ films. Nanoscale.

[36] Yim C, O’Brien M, McEvoy N, Winters S, Mirza I, Lunney JG, Duesberg GS (2014). Investigation of the optical properties of MoS_2_ thin films using spectroscopic ellipsometry. Appl. Phys. Lett..

[37] Bai L, Cai X, Lu J, Li L, Zhong S (2018). Surface and interface engineering in Ag_2_S@MoS_2_ core–shell nanowire heterojunctions for enhanced visible photocatalytic hydrogen production. ChemCatChem.

[38] Zuo P, Jiang L, Li X, Li B, Ran P (2018). Metal (Ag, Pt)–MoS_2_ hybrids greenly prepared through photochemical reduction of femtosecond laser pulses for SERS and HER. ACS Sustain. Chem. Eng..

[39] Zhu J, Wang Z, Yu H, Li N, Zhang J (2017). Argon plasma induced phase transition in monolayer MoS_2_. J. Am. Chem. Soc..

[40] Major JD, Al Turkestani M, Bowen L, Brossard M, Li C (2016). In-depth analysis of chloride treatments for thin-film CdTe solar cells. Nat. Commun..

[41] Eda G, Yamaguchi H, Voiry D, Fujita T, Chen M, Chhowalla M (2011). Photoluminescence from chemically exfoliated MoS_2_. Nano Lett..

[42] Lin WH, Chiu YH, Shao PW, Hsu YJ (2016). Metal-particle-decorated ZnO nanocrystals: photocatalysis and charge dynamics. ACS Appl. Mater. Interfaces.

[43] Chen YC, Pu YC, Hsu YJ (2012). Interfacial charge carrier dynamics of the three-component In_2_O_3_ − TiO_2_ − Pt heterojunction system. J. Phys. Chem. C.

[44] Jalali M, Moakhar RS, Abdelfattah T, Filine E, Mahshid SS, Mahshid S (2020). Nanopattern-assisted direct growth of peony-like 3D MoS2/Au composite for nonenzymatic photoelectrochemical sensing. ACS Appl. Mater. Interfaces.

[45] Patra KK, Gopinath CS (2016). Bimetallic and plasmonic Ag–Au on TiO_2_ for solar water splitting: an active nanocomposite for entire visible-light-region absorption. ChemCatChem.

[46] Burda C, Chen X, Narayanan R, El-Sayed MA (2005). Chemistry and properties of nanocrystals of different shapes. Chem. Rev..

[47] Lee H, Deshmukh S, Wen J, Costa VZ, Schuder JS (2019). Layer-dependent interfacial transport and optoelectrical properties of MoS2 on ultraflat metals. ACS Appl. Mater. Interfaces.

[48] Y.J. Yu, Y. Zhao, S. Ryu, L.E. Brus, K.S. Kim, P. Kim, Tuning the graphene work function by electric field effect. Nano Lett. **9**(10), 3430–3434 (2009). 10.1021/nl901572a 10.1021/nl901572a 19719145

[49] Cao D, Luo W, Feng J, Zhao X, Lia Z, Zou Z (2014). Cathodic shift of onset potential for water oxidation on a Ti^4+^ doped Fe_2_O_3_ photoanode by suppressing the back reaction. Energy Environ. Sci..

[50] Khan SUM, Al-Shahry M, Ingler WB (2002). Efficient photochemical water splitting by a chemically modified n-TiO_2_. Science.

[51] Xu X, Zhou G, Dong X, Hu J (2017). Interface band engineering charge transfer for 3D MoS_2_ photoanode to boost photoelectrochemical water splitting. ACS Sustain. Chem. Eng..

[52] Pi Y, Li Z, Xu D, Liu J, Li Y (2017). 1T-phase MoS_2_ nanosheets on TiO_2_ nanorod arrays: 3D photoanode with extraordinary catalytic performance. ACS Sustain. Chem. Eng..

[53] Pan Q, Zhang C, Xiong Y, Mi Q, Li D (2018). Boosting charge separation and transfer by plasmon-enhanced MoS_2_/BiVO_4_ p–n heterojunction composite for efficient photoelectrochemical water splitting. ACS Sustain. Chem. Eng..

[54] Zeng L, Li X, Fan S, Zhang M, Yin Z, Tadé M, Liu S (2019). Photo-driven bioelectrochemical photocathode with polydopamine-coated TiO_2_ nanotubes for self-sustaining MoS_2_ synthesis to facilitate hydrogen evolution. J. Power Sources.

[55] Moniz SJA, Shevlin SA, Martin DJ, Guo Z-X, Tang J (2015). Visible-light driven heterojunction photocatalysts for water splitting—a critical review. Energy Environ. Sci..

[56] Jiang C, Moniz SJA, Wang A, Zhang T, Tang J (2017). Photoelectrochemical devices for solar water splitting–materials and challenges. Chem. Soc. Rev..

