# Supplementary material for: Plasmonic Ag-Decorated Few-Layer MoS2 Nanosheets Vertically Grown on Graphene for Efficient Photoelectrochemical Water Splitting
Source: Nanomicro Lett. 2020 Aug 25;12:172. doi: 10.1007/s40820-020-00512-3 (PMC7770824; doi:10.1007/s40820-020-00512-3)
Supplement: Supplementary file 1 — Supplementary material 1 (PDF 798 kb) [file 40820_2020_512_MOESM1_ESM.pdf]

Supporting Information for

Plasmonic Ag-Decorated Few-Layer MoS_2 Nanosheets Vertically Grown on Graphene for Efficient Photoelectrochemical Water Splitting

Dong-Bum Seo[1], Tran Nam Trung[1], Dong-Ok Kim[1], Duong Viet Duc[1], Sungmin Hong[2], Youngku Sohn[2], Jong-Ryul Jeong[1], Eui-Tae Kim[1,] *

[1]Department of Materials Science & Engineering, Chungnam National University, Daejeon 34134, Republic of Korea

[2]Department of Chemistry, Chungnam National University, Daejeon 34134, Republic of Korea

*Corresponding author. E-mail: etkim@cnu.ac.kr (Eui-Tae Kim)

Supplementary Figures and Table

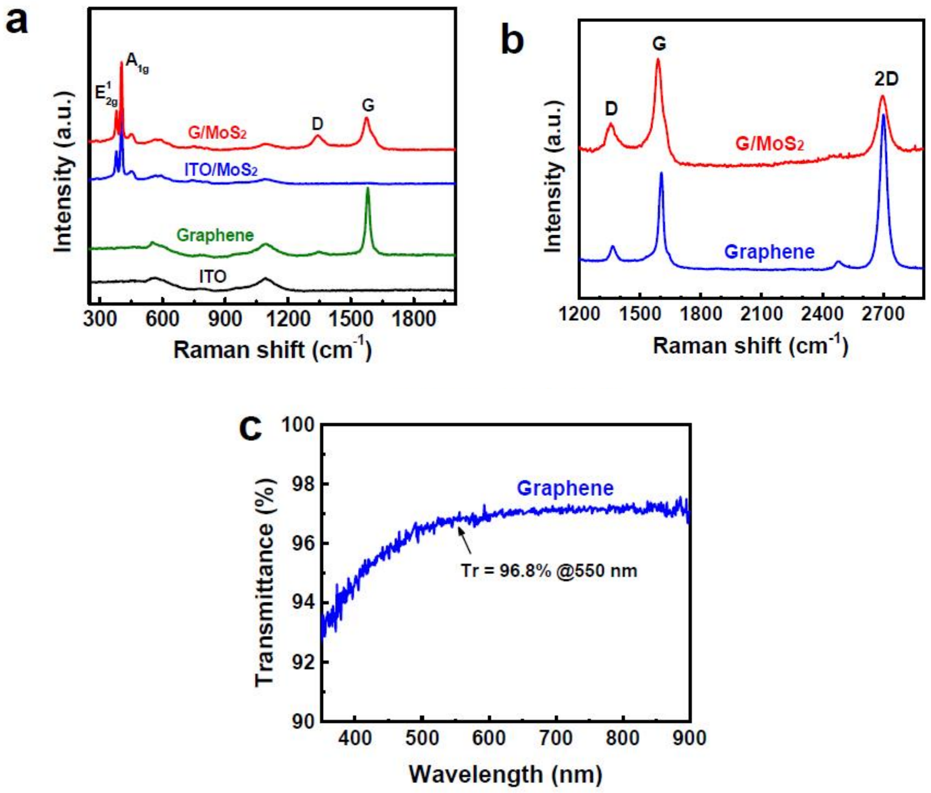

Fig. S1 (**a**) Raman spectra of ITO, pristine graphene, ITO/MoS2, and G/MoS2. (**b**) Graphene-region Raman spectra of pristine graphene and G/MoS2. (**c**) UV–Vis absorption spectrum of pristine graphene, exhibiting light transmittance of 96.8% at 550 nm

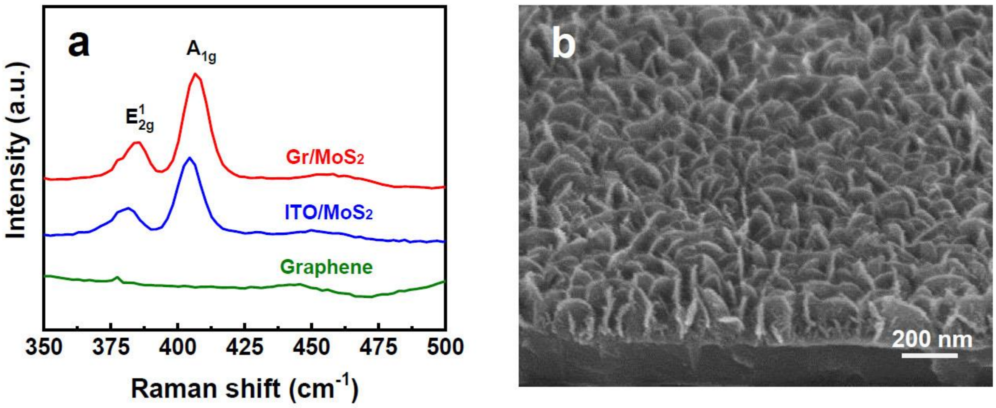

Fig. S2 (**a**) Raman spectra of pristine graphene, ITO/MoS_2, and G/MoS_2. (**b**) SEM images of fewlayer MoS_2 nanosheets grown on ITO (ITO/MoS_2)

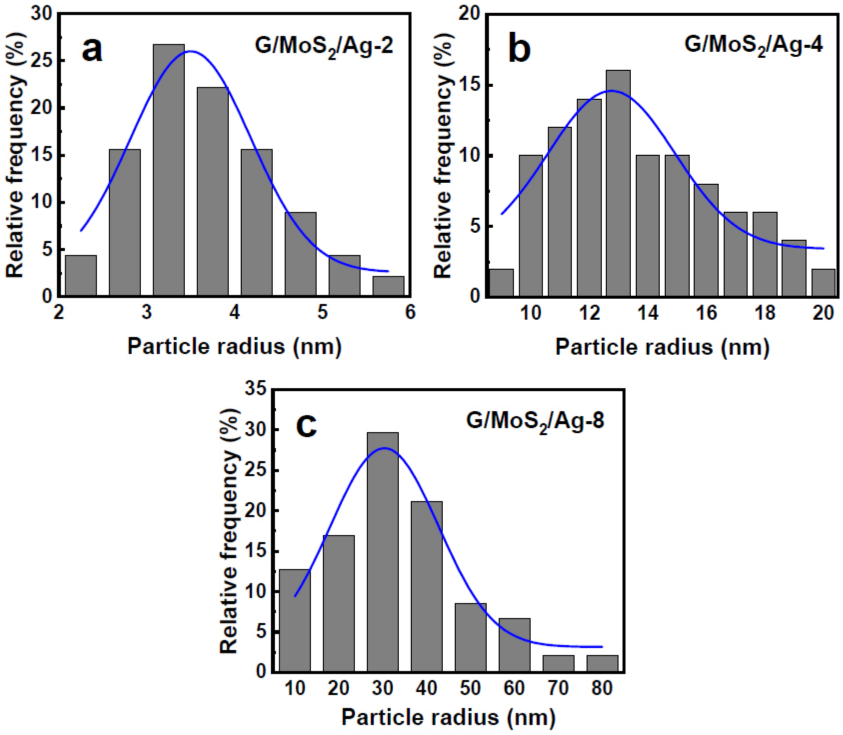

Fig. S3 Ag NP size distribution of (**a**) G/MoS2/Ag-2, (**b**) G/MoS2/Ag-4, and (**c**) G/MoS2/Ag-8

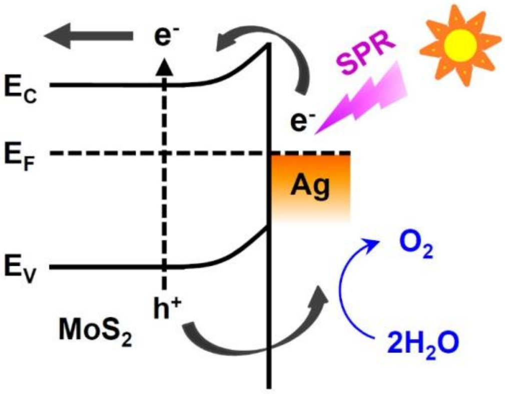

Fig. S4 Energy band diagram of the Schottky junction of MoS_2/Ag

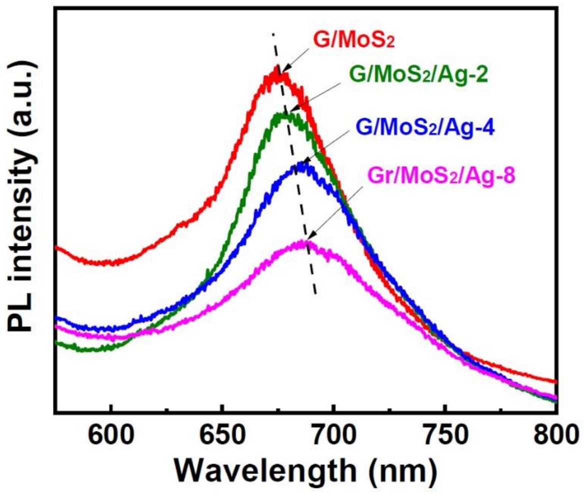

Fig. S5 PL spectra of G/MoS2, G/MoS2/Ag-2, G/MoS2/Ag-4, and G/MoS2/Ag-8

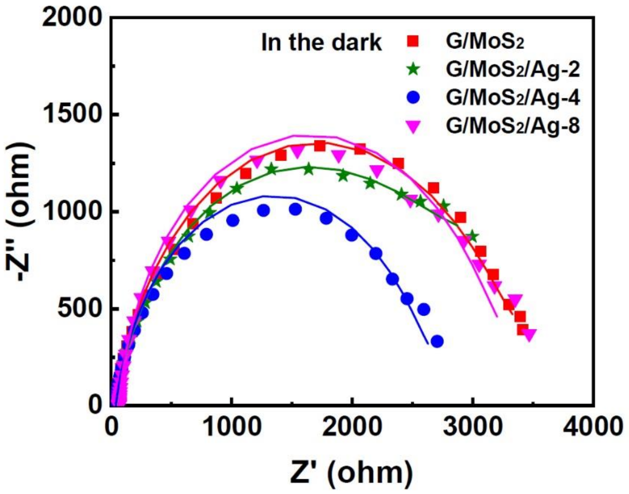

Fig. S6 Nyquist plots of G/MoS2, G/MoS2/Ag-2, G/MoS2/Ag-4, and G/MoS2/Ag-8 in the dark

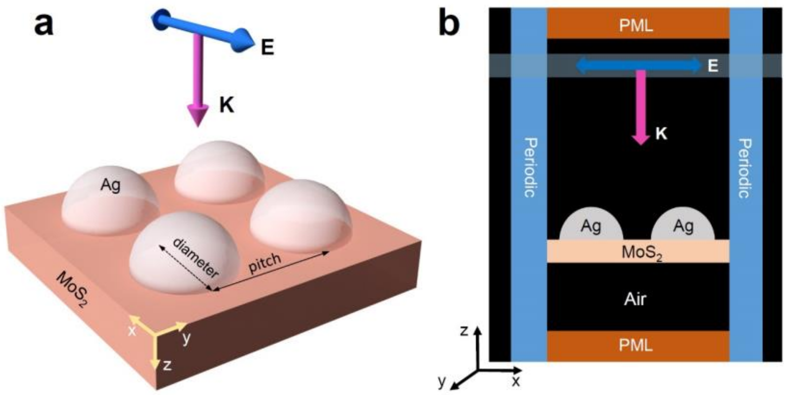

Fig. S7 (**a**) Schematic representation of the formulated structure for FDTD simulations. The Ag NPs on a three-layer-thick MoS2 substrate is irradiated by a plane-wave source with the propagation vector in the *z*-direction and the E-field oscillating along the *x*-axis. (**b**) Real simulation environment with applied periodic boundary conditions

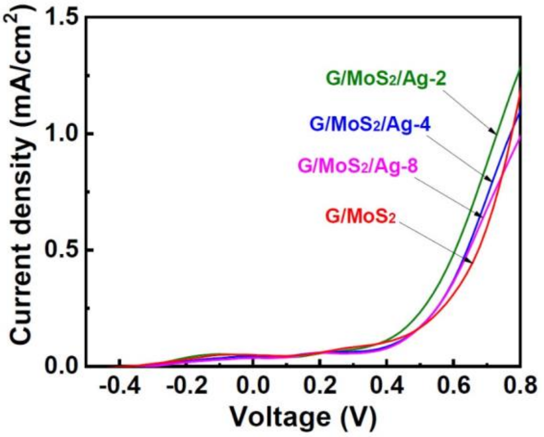

Fig. S8 Dark current density–potential curves of PEC cells with various working electrodes (G/MoS_2, G/MoS_2/Ag-2, G/MoS_2/Ag-4, and G/MoS_2/Ag-8)

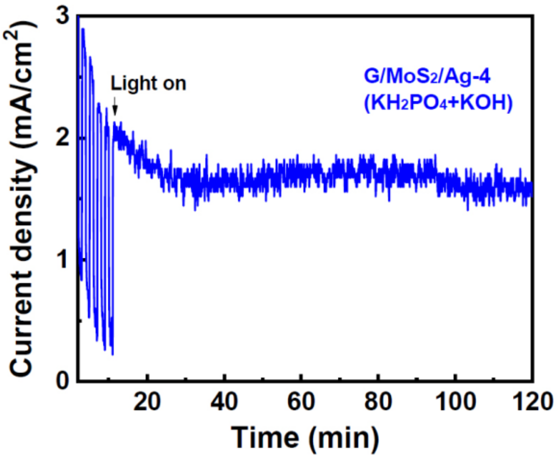

Fig. S9 Repeated photocurrent–time measurement for G/MoS2/Ag-4 in 0.3 M KH2PO4 + 0.3 M KOH solution after a month

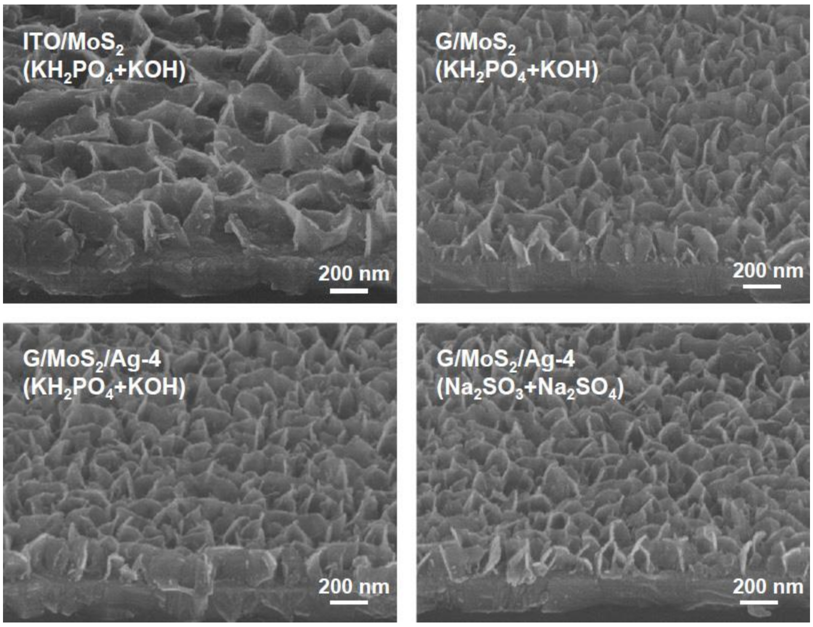

Fig. S10 SEM images of ITO/MoS2, G/MoS2, and G/MoS2/Ag-4 after PEC measurement for 1 h

Table S1 R_{ct} and R_s values of EIS analysis in the dark and under illumination

Samples	Dark		Illumination		R_{ct} (dark)/R_{ct} (photo)
	R_{ct} [Ω]	R_s [Ω]	R_{ct} [Ω]	R_s [Ω]	
ITO/MoS_2	4236	43.7	2766	45.5	1.53
G/MoS_2	3264	40.1	1959	40.2	1.67
G/MoS_2/Ag-2	2834	41.7	1780	41.7	1.59
G/MoS_2/Ag-4	2572	40.2	1284	44.2	2.00
G/MoS_2/Ag-8	3110	66.8	1947	48.3	1.60
